# Supplementary material for: Potentials and limitations in the application of Convolutional Neural Networks for mosquito species identification using wing images
Source: PLoS Comput Biol. 2025 Sep 5;21(9):e1013435. doi: 10.1371/journal.pcbi.1013435 (PMC12412992; doi:10.1371/journal.pcbi.1013435)
Supplement: S1 File — Table A. Glossary. Table B. Hyperparameters. Table C. Taxa Labels and Taxonomic IDs. Table D - F. Robustness experiment data distribution. Table G. Metrics. Fig A. Examples images from the Feasibility Study. (DOCX) [file pcbi.1013435.s001.docx]

**Supporting information**

## **S1 Table A:** Glossary

Table A: Abbreviations used in the manuscript.

| **Abbreviation** | **Definition** |
| --- | --- |
| CI95% | 95% confidence interval |
| CNN | Convolutional Neural Network |
| CLAHE | Contrast Limited Adaptive Histogram Equalisation |
| GradCam | Gradient-weighted Class Activation Mapping |
| ML | Machine Learning |
| UMAP | Uniform Manifold Approximation and Projection |

**S1 Table B:** Hyperparameters

Table B: Hyperparameter Settings for Training of Main Classifier.

| **Parameters** | **Values** |
| --- | --- |
| Model Architecture | EfficientNetB0 |
| Image Resolution | 192x384 |
| Batch Size | 32 |
| Learning Rate | 0.0005 |
| Learning Rate Schedule | CosineDecay |
| Learning Rate Warmup Fraction | 0.2 |
| Images per Epoch | 14888 |
| Epochs | 32 |
| Dropout Probability | 0.5 |
| ISONoise | (0.01, 0.05), P=0.5 |
| PlanckianJitter | P=0.5 |
| ImageCompression | (75, 100), P=0.25 |
| Defocus | (1,3), P=0.25 |
| RandomGamma | (80,120), P=0.25 |
| MotionBlur | (3,3), P=0.25 |
| Downscale | (0.75, 1), P=0.25 |
| ColorJitter | Brightness=0.2, Contrast=0.2, Saturation=0.2, Hue=0.2, P=0.5 |
| ChannelDropout | (1,1), P=0.25 |
| MultiplicativeNoise | (0.9, 1.1), P=0.25 |

**S1 Table C:** Taxa Labels and Taxonomic IDs

Table C: Taxon Label and ITIS Taxonomic Serial Number

| **Taxon Label** | **Scientific Name** | **ITIS Taxonomic Serial Number** |
| --- | --- | --- |
| *Aedes aegypti* | *Ae. aegypti* | 126240 |
| *Aedes albopictus* | *Ae. albopictus* | 126244 |
| *Aedes annulipes-group* | *Ae. annulipes*  *Ae. cantans*  *Ae. excrucians*  *Ae. flavescens*  *Ae. riparius* | 1154465  1154483  126288  126298  126357 |
| *Aedes caspius* | *Ae. caspius* | 1154484 |
| *Aedes cataphylla* | *Ae. cataphylla* | 126261 |
| *Aedes cinereus-geminus pair* | *Ae. cinereus*  *Ae. geminus* | 126264  1151864 |
| *Anopheles claviger-petragani pair* | *An. claviger*  *An. petragnani* | 1151372  1153041 |

| *Aedes communis-punctor pair* | *Ae. communis*  *Ae. punctor* | 126267  126351 |
| --- | --- | --- |
| *Aedes japonicus* | *Ae. japonicus japonicus* | 1155106 |
| *Aedes koreicus* | *Ae. koreicus* | 1154365 |
| *Anopheles maculipennis s.l.* | *An. maculipennis*  *An. atroparvus*  *An. daciae*  *An. messeae* | 1152508  1150988  1151496  1152641 |
| *Culex modestus* | *Cx. modestus* | 1152694 |
| *Culiseta morsitans- fumipennis pair* | Cs. morsitans  Cs. fumipennis | 126446  1151807 |
| *Culex torrentium – pipiens s.l. pair* | *Cx. torrentium*  *Cx. quinquefasciatus*  *Cx. pipiens pipiens*  *Cx. pipiens molestus* | 1153744  126490  1155008  126542 |
| *Coquillettidia richiardii* | *Cq. richiardii* | 1153308 |
| *Aedes rusticus* | *Ae. rusticus* | 1154691 |
| *Anopheles stephensi* | *An. stephensi* | 1153588 |
| *Aedes sticticus* | *Ae. sticticus* | 126368 |
| *Aedes vexans* | *Ae. vexans* | 126403 |
| *Culex vishnui-group* | *Cx. pseudovishnui*  *Cx. tritaeniorhynchus*  *Cx. vishnui* | 1153177  1153780  1153880 |
| *Other* | *An. plumbeus*  *An. moucheti*  *An. coustani*  *An. paludis*  *An. nili s.l.*  *An. gambiae s.l.*  *Ae. geniculatus*  *Ae. rossicus*  *Cx. territans*  *Cx. hortensis*  *Cs. annulata*  *Cs. subochrea*  *Ae. intrudens*  *Ae. pulcritarsis*  *An. hyrcanus*  *Cq. buxtoni*  *Uranotaenia unguiculata*  *An. sinensis*  *An. tessellatus*  *Armigeres durhami*  *Armigeres subalbatus*  *Cq. crassipes*  *Cx. brevipalpis*  *Cx. fuscocephala*  *Cx. gelidus*  *Cx. nigropunctatus*  *Cx. orientalis*  *Mansonia uniformis*  *Toxorhynchites splendens*  *Ae. amesii*  *Ae. ostentatio*  *Ae. thailandensis*  *Ae. vittatus*  *An. aconitus*  *An. epiroticus*  *Armigeres jugraensis*  *Cx. bitaeniorhynchus*  *Mansonia indiana* | 1153093  1152717  1151446  1152954  1152814  125987  1154048  1153344  126501  1152056  126432  126454  126319  1154553  1152077  1151217  1153804  1153505  1153703  1151617  1153612  1151455  1151179  1151827  1151862  1152807  1152904  1153808  125934  1154393  1154591  1154593  1154294  1150727  1151669  1152197  1151145  1152119 |

**S1 Table D:** Robustness Experiment Data Distribution - Olympus Device Experiment

Table D: Distribution of Dataset for Device Experiment where models were only trained on microscope images. Five models were trained on all folds except one and the testing folds (containing all images of the other device). Models were then evaluated on the left out fold and the testing set.

| **Fold** | **Device** | **Taxa Label** | **count** |
| --- | --- | --- | --- |
| testing | macrolens + iphone se | Ae. aegypti | 386 |
| testing | macrolens + iphone se | Ae. japonicus | 378 |
| testing | macrolens + iphone se | Ae. albopictus | 374 |
| testing | macrolens + iphone se | Ae. koreicus | 368 |
| 0 | olympus sz61 + olympus dp23 | Ae. aegypti | 80 |
| 0 | olympus sz61 + olympus dp23 | Ae. japonicus | 77 |
| 0 | olympus sz61 + olympus dp23 | Ae. albopictus | 77 |
| 0 | olympus sz61 + olympus dp23 | Ae. koreicus | 76 |
| 1 | olympus sz61 + olympus dp23 | Ae. japonicus | 78 |
| 1 | olympus sz61 + olympus dp23 | Ae. aegypti | 77 |
| 1 | olympus sz61 + olympus dp23 | Ae. koreicus | 77 |
| 1 | olympus sz61 + olympus dp23 | Ae. albopictus | 76 |
| 2 | olympus sz61 + olympus dp23 | Ae. aegypti | 78 |
| 2 | olympus sz61 + olympus dp23 | Ae. albopictus | 76 |
| 2 | olympus sz61 + olympus dp23 | Ae. japonicus | 75 |
| 2 | olympus sz61 + olympus dp23 | Ae. koreicus | 72 |
| 3 | olympus sz61 + olympus dp23 | Ae. aegypti | 79 |
| 3 | olympus sz61 + olympus dp23 | Ae. koreicus | 76 |
| 3 | olympus sz61 + olympus dp23 | Ae. japonicus | 73 |
| 3 | olympus sz61 + olympus dp23 | Ae. albopictus | 71 |
| 4 | olympus sz61 + olympus dp23 | Ae. aegypti | 80 |
| 4 | olympus sz61 + olympus dp23 | Ae. japonicus | 77 |
| 4 | olympus sz61 + olympus dp23 | Ae. albopictus | 73 |
| 4 | olympus sz61 + olympus dp23 | Ae. koreicus | 73 |

**S1 Table E:** Robustness Experiment Data Distribution - Phone Device Experiment

Table E: Distribution of Dataset for Device Experiment where models were only trained on smartphone images. Five models were trained on all folds except one and the testing folds (containing all images of the other device). Models were then evaluated on the left out fold and the testing set.

| **Fold** | **Device** | **Taxa Label** | **count** |
| --- | --- | --- | --- |
| testing | olympus sz61 + olympus dp23 | *Ae. aegypti* | 394 |
| testing | olympus sz61 + olympus dp23 | *Ae. japonicus* | 380 |
| testing | olympus sz61 + olympus dp23 | *Ae. koreicus* | 374 |
| testing | olympus sz61 + olympus dp23 | *Ae. albopictus* | 373 |
| 0 | macrolens + iphone se | *Ae. aegypti* | 79 |
| 0 | macrolens + iphone se | *Ae. japonicus* | 79 |
| 0 | macrolens + iphone se | *Ae. koreicus* | 77 |
| 0 | macrolens + iphone se | *Ae. albopictus* | 76 |
| 1 | macrolens + iphone se | *Ae. japonicus* | 77 |
| 1 | macrolens + iphone se | *Ae. koreicus* | 77 |
| 1 | macrolens + iphone se | *Ae. albopictus* | 76 |
| 1 | macrolens + iphone se | *Ae. aegypti* | 75 |
| 2 | macrolens + iphone se | *Ae. aegypti* | 77 |
| 2 | macrolens + iphone se | *Ae. albopictus* | 75 |
| 2 | macrolens + iphone se | *Ae. japonicus* | 75 |
| 2 | macrolens + iphone se | *Ae. koreicus* | 71 |
| 3 | macrolens + iphone se | *Ae. aegypti* | 78 |
| 3 | macrolens + iphone se | *Ae. koreicus* | 74 |
| 3 | macrolens + iphone se | *Ae. albopictus* | 72 |
| 3 | macrolens + iphone se | *Ae. japonicus* | 71 |
| 4 | macrolens + iphone se | *Ae. aegypti* | 77 |
| 4 | macrolens + iphone se | *Ae. japonicus* | 76 |
| 4 | macrolens + iphone se | *Ae. albopictus* | 75 |
| 4 | macrolens + iphone se | *Ae. koreicus* | 69 |

**S1 Table F:** Robustness Experiment Data Distribution - Bias Experiment

Table F:Distribution of Dataset for Bias Experiments. Five models were trained on all folds except one and the testing folds. Models were then evaluated on the left out fold and the testing set.

| **Fold** | **Device** | **Taxa Label** | **count** |
| --- | --- | --- | --- |
| testing | macrolens + iphone se | *Ae. aegypti* | 386 |
| testing | macrolens + iphone se | *Ae. koreicus* | 368 |
| testing | olympus sz61 + olympus dp23 | *Ae. japonicus* | 380 |
| testing | olympus sz61 + olympus dp23 | *Ae. albopictus* | 373 |
| 0 | macrolens + iphone se | *Ae. japonicus* | 79 |
| 0 | macrolens + iphone se | *Ae. albopictus* | 76 |
| 0 | olympus sz61 + olympus dp23 | *Ae. aegypti* | 80 |
| 0 | olympus sz61 + olympus dp23 | *Ae. koreicus* | 76 |
| 1 | macrolens + iphone se | *Ae. japonicus* | 77 |
| 1 | macrolens + iphone se | *Ae. albopictus* | 76 |
| 1 | olympus sz61 + olympus dp23 | *Ae. koreicus* | 77 |
| 1 | olympus sz61 + olympus dp23 | *Ae. aegypti* | 77 |
| 2 | macrolens + iphone se | *Ae. albopictus* | 75 |
| 2 | macrolens + iphone se | *Ae. japonicus* | 75 |
| 2 | olympus sz61 + olympus dp23 | *Ae. aegypti* | 78 |
| 2 | olympus sz61 + olympus dp23 | *Ae. koreicus* | 72 |
| 3 | macrolens + iphone se | *Ae. albopictus* | 72 |
| 3 | macrolens + iphone se | *Ae. japonicus* | 71 |
| 3 | olympus sz61 + olympus dp23 | *Ae. aegypti* | 79 |
| 3 | olympus sz61 + olympus dp23 | *Ae. koreicus* | 76 |
| 4 | macrolens + iphone se | *Ae. japonicus* | 76 |
| 4 | macrolens + iphone se | *Ae. albopictus* | 75 |
| 4 | olympus sz61 + olympus dp23 | *Ae. aegypti* | 80 |
| 4 | olympus sz61 + olympus dp23 | *Ae. koreicus* | 73 |

**S1 Text A:** Metrics

The accuracy and F1-score were used as performance metrics (Table 1). To adapt the performance metrics for the imbalanced multilabel classification, we used balanced accuracy and macro F1 score. We assessed the interrater reliability, by utilizing the Fleiss' Kappa, which measures the agreement between multiple raters when assigning categorical ratings to a fixed number of items (Shrout and Fleiss, 1979). To calculate the metrics, we utilised the Python libraries scikit-learn and statsmodels (Pedregosa et al., 2011; Seabold and Perktold, 2010). The code which was used to analyse the data can be found on the associated GitHub repository: <https://github.com/KNolte19/MosquitoWingClassifier_publication>

**S1 Table G**: Metrics

Table F: Metrics and their definition which were used to assess the performance of the classification system. TP: True positives, TN: True negatives, FP: False positives, FN: False negatives.

| **Metric** | **Equation** |
| --- | --- |
| *Accuracy* | $\frac{TP+TN}{TP+FP+TN+FN}$ |
| *Precision* | $\frac{TP}{TP+FP}$ |
| *Recall* | $\frac{TP}{TP+FN}$ |
| *F1 Score* | $2*\frac{Precision*Recall}{Precision+Recall}$ |

**S1 Fig A:** Example images from the Feasibility study

| 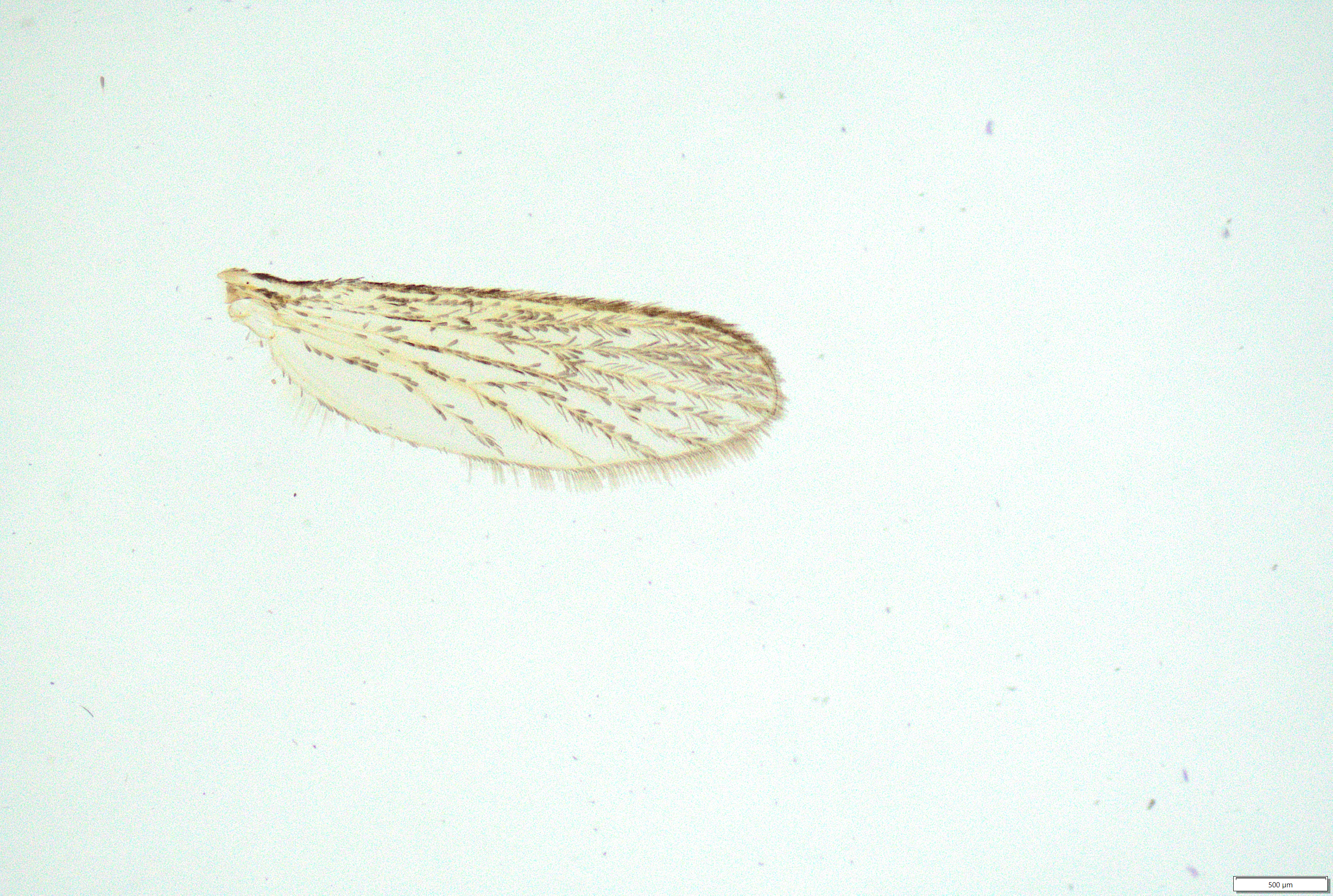 | 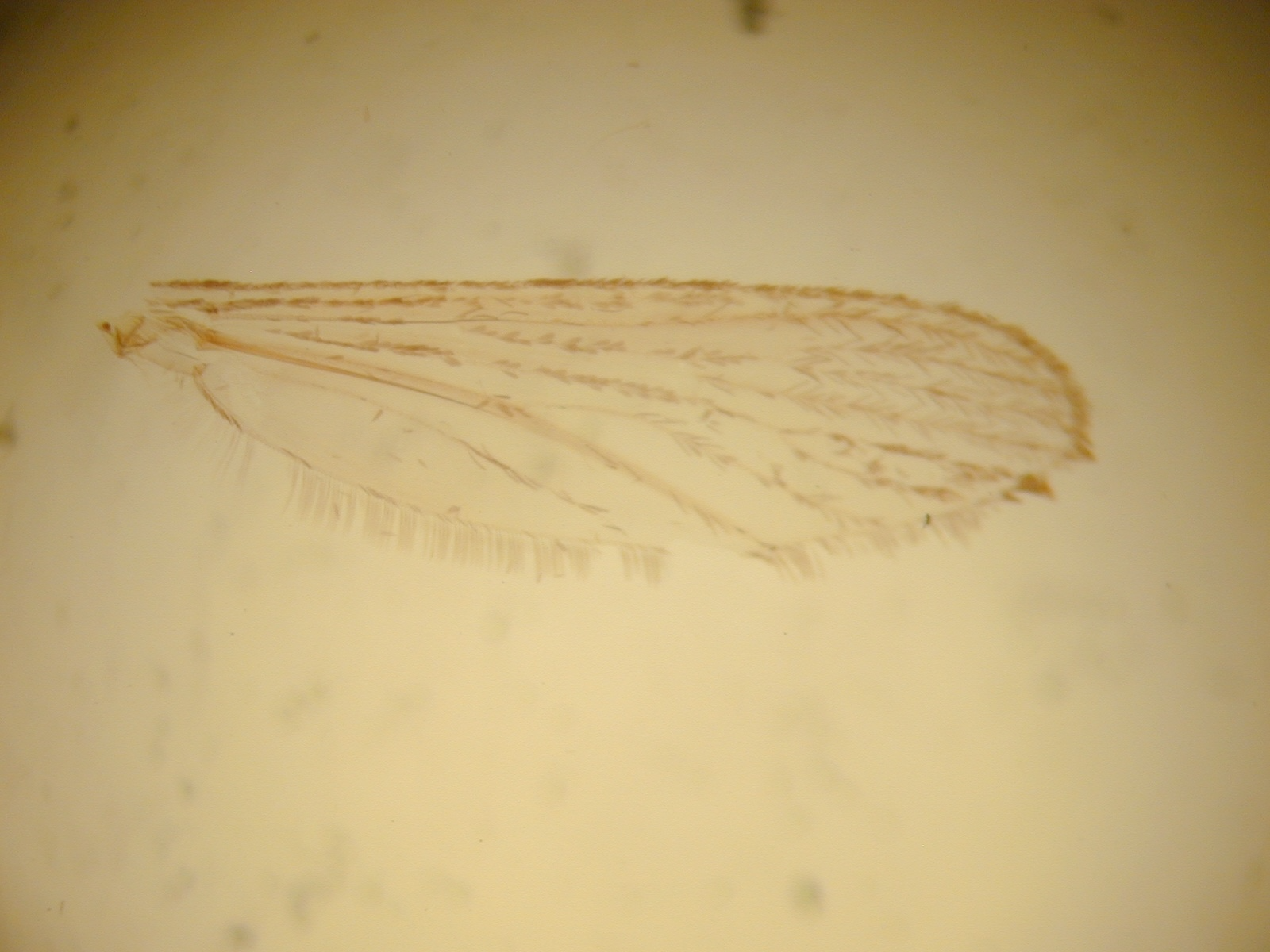 |
| --- | --- |
| **Olympus SZ61 +  Olympus DP23** | **Zeiss Stemi 2000-C stereomicroscope + Nikon Coolpix P950** |

**References**

Pedregosa, F., Varoquaux, G., Gramfort, A., Michel, V., Thirion, B., Grisel, O., Blondel, M., Prettenhofer, P., Weiss, R., Dubourg, V., Vanderplas, J., Passos, A., Cournapeau, D., 2011. Scikit-learn: Machine Learning in Python. J. Mach. Learn. Res. 12, 2825–2830.

Seabold, S., Perktold, J., 2010. Statsmodels: Econometric and Statistical Modeling with Python. Presented at the Python in Science Conference, Austin, Texas, pp. 92–96. https://doi.org/10.25080/Majora-92bf1922-011

Shrout, P.E., Fleiss, J.L., 1979. Intraclass correlations: Uses in assessing rater reliability. Psychol. Bull. 86, 420–428. https://doi.org/10.1037/0033-2909.86.2.420
