# Supplementary material for: Potentials and limitations in the application of Convolutional Neural Networks for mosquito species identification using wing images
Source: PLoS Comput Biol. 2025 Sep 5;21(9):e1013435. doi: 10.1371/journal.pcbi.1013435 (PMC12412992; doi:10.1371/journal.pcbi.1013435)
Supplement: Attachment — Submitted filename: Respone to Reviewers.docx [file pcbi.1013435.s002.docx]

Dear Editors,

Thanks for the invitation to respond to the reviewer comments. Below you find all comments in grey and our author response in **black**. In the revised manuscript all new changes are indicated in yellow.

With kind regards

Kristopher Nolte

Reviewers' comments:

Reviewer's Responses to Questions

**Reviewer #1**: The authors present a manuscript detailing the application of convolutional neural networks (CNNs) for identifying mosquito species using wing images. The introduction effectively highlights the need for a reliable and user-friendly system that can be deployed in resource-limited regions. The authors provide a comprehensive description of the preprocessing of wing images, the training of their CNNs (based on the EfficientNet architecture), and the results of testing these models. The reported accuracy is impressively high, rarely dropping below 97% (Figure 4). Additionally, the authors commendably make their code freely available, although I encountered some difficulties in executing it (see below).

However, I have several concerns regarding the manuscript in its current form. Firstly, the high accuracy of the models suggests potential overfitting to the training data. The authors acknowledge this issue in Figure 5, noting that models performing well on familiar data may struggle with new data. To demonstrate the models' practical utility, Section 4.4, which describes a feasibility study, needs substantial expansion. Furthermore, the feasibility study used the same imaging system (Olympus SZ61) as most of the training data; it would be beneficial to explore how the models perform with different imaging systems.

We agree that the original feasibility study had limitations due to the use of the same imaging device as in the training data. Therefore, we have extended the study by including a new imaging setup: all images were re-captured by a single participant using a Zeiss Stemi 2000-C stereomicroscope paired with a Nikon Coolpix P950. This imaging system was not represented in the original dataset. We report that the models achieved a high and comparable accuracy of 95.91% (95% CI: 94.96%–96.85%) on this new dataset. We have updated the manuscript accordingly in line 290-293, 396-397 and 400-407 to reflect this improvement to our study.

My most significant concern is the manuscript's similarity to two previously published works by a similar group of authors from last year:
* <https://parasitesandvectors.biomedcentral.com/articles/10.1186/s13071-024-06459-3>
* <https://www.nature.com/articles/s41598-024-53631-x>.
Both papers describe similar approaches using CNNs to identify mosquito species based on wing images, with one even utilizing the same EfficientNet architecture. The authors should acknowledge these previous works more directly in their manuscript (they are currently only cited as the source of their images) and clarify how the current study represents a significant advancement. The primary difference appears to be the inclusion of a larger number of mosquito species. The introduction should be revised to clearly state that this work builds upon previously published research by the same authors. Additionally, the introduction lacks references to the shortcomings of other published works. Overall, a much stronger case needs to be made for the novelty and significance of the current study.

We agree that we could have been more explicit in linking this manuscript to our prior studies. In response, we have revised the introduction to clearly explain how this work builds on our previous research. While earlier publications focused on establishing proof-of-concept models, this study emphasizes practical deployment and robustness. Specifically, we now address three critical challenges: scalability to more species, robustness to imaging variation, and usability for field researchers. In the end we present a robust, deployable application designed with real-world constraints in mind. We have updated the manuscript to better reflect these contributions and clearly differentiate it from our prior work.

We changed the manuscript in line 75, 91-92,102 – 117, 121-122, 134-135, 141-143 and 229-231.

Other points:
* Discussion of the labelling strategy for training data in the methods is a little confusing and Figure 1 is not very informative. I think a table summarising the exact composition of all data used for training, validation and testing would be helpful

We completely overhauled chapter 3.1 and put emphasis on the understandability of our description. As a table containing the information would be very large, we refer the reader to the Git repository and the newly added supplementary table S4 regarding more detailed information on the label strategy and number of images. We modified the manuscript in line 121 – 157.

* The distinction between what constitutes preprocessing and augmentation could be clearer. Augmentation is for training purposes only, but preprocessing is always necessary. For example, this statement (lines 162-163) is confusing: "During training the augmentation is used to increase the variance of the training set, during deployment the augmentation is used for test-time augmentation, only during testing this step is omitted"

We have revised the relevant section in the manuscript to more clearly delineate preprocessing (applied in training, testing and deployment) from augmentation (applied only during training and deployment as test-time augmentation). The updated phrasing eliminates ambiguity and improves the overall clarity of our methodology:

line 188 – 192 and line 196.

* The Pre-processing seems quite involved - I would be surprised if it works reliably with unseen data. Have the authors tried training the classifier without the preprocessing step? Because this might generalise better?

In our initial experiments, we trained models without preprocessing, but we observed a decline in performance on data from unseen imaging devices. We reported this in one of our previous publications (11) where we described that CNN models tend to overfit to device-specific features. This limitation motivated the development of the here presented preprocessing pipeline. To investigate whether our pipeline really was able to reduce this bias we designed the robustness experiments and the results support this: models trained without preprocessing fail to generalize to new devices, while those with preprocessing perform significantly better (Figure 5).

Regarding the point that the preprocessing might not work with novel imaging setups we did some experiments and found the preprocessing to be very robust. In our study, we evaluated images captured via Phone (iPhone SE +.Apexel-24XMH), an older stereomicroscope-digital camera systems (Zeiss Stemi 2000-C stereomicroscope + Nikon Coolpix P950) and more sophisticated system (Olympus SZ61 + Olympus DP23) which all results in good and similar performance even when images looked very different (we show you some examples below). While the preprocessing pipeline consists of multiple steps each step is not very vulnerable to changes in the image depiction and does not require the finetuning of parameters. We therefore expect that if the user takes a reasonable image following our simple guidelines, the preprocessing pipeline will produce images in good quality.

|  |  |  |
| --- | --- | --- |
| **iPhone SE +. Apexel x20** | **Olympus SZ61 + Olympus DP23** | **Zeiss Stemi 2000-C stereomicroscope + Nikon Coolpix P950** |

* No real explanation is offered as to why EfficientNet was used? Did the authors try any other architectures? Has anyone else previously published other models used for similar purposes?

Indeed, we experimented with various architectures including InceptionNet, ResNet, MobileNet, DenseNet, and MobileViT. While several of these models performed comparably, EfficientNet offered a favorable trade-off between accuracy, parameter efficiency, and input size flexibility. These practical advantages motivated our choice. We have now added this rationale to the manuscript for clarity in line 212-214.

* Essentially, 5 different models were trained and the results presented show the average result returned by the 5 models. But how much variability was there across the five models?

To address the reviewer’s point on variability, we have now included the minimum and maximum balanced accuracy values across the five trained models in line 317-318, in addition to the 95% confidence interval already reported.

* Lines 210 - 214 are confusing. Again, a table listing the details of the training data would be more useful here.

* Lines 215 - 222: I have to be honest, I'm getting a little lost here - I have no idea (a) what the aim is here or (b) what is being used for training data and what is being used for testing. We seem to have gone off on a tangent.

* In the "Robustness experiments" section, is the training data referred to here completely distinct from that referenced earlier? Or is it a subset? I ask, because the description of the dataset seems to closely match characteristics shown in Fig 1.

* There are multiple references throughout the paper to different training sets - it is not totally clear what training data was actually used to generate each of the presented results. I would suggest having a table describing exactly what data was used for training for each Figure.

We thank the reviewer for pointing out the confusion regarding the training/testing setup in the robustness experiments. We recognize that this section was not as clear as it could be. The robustness experiments used a well-controlled subset of the main dataset, sourced from a previously published study. To clarify this, we have added a dedicated table outlining the dataset compositions used for each experiment in Supplementary S4. We also completely revised the corresponding sections in the manuscript to improve the explanation of these experiments in line 225-262.

* It's great that the authors have made code openly-available, but there is little in the way of installation assistance in the Github repo. I tried setting up the necessary python environment to run the notebooks provided (<https://github.com/KNolte19/MosquitoWingClassifier_publication>), but even after correcting some apparent typos in the requirements file, the pip installation still failed:
ERROR: Could not find a version that satisfies the requirement tensorflow-macos==2.14.0 (from versions: none)
ERROR: No matching distribution found for tensorflow-macos==2.14.0

We acknowledge the challenges encountered and have since restructured the GitHub repository to simplify installation and reproducibility.

**Reviewer #2**: In this manuscript, the authors developed methods to identify mosquito species from wing images by a CNN model trained with a large curated database. They tested the robustness of identification under different imaging devices and built a data augmentation pipeline to improve model's generalization capability. Authors also tested model's performance in a feasibility study.

The data augmentation successfully reduced the identification errors introduced by device differences, but when the strong correlation between species and devices existed, generalization was difficult.

While many accurate models for mosquito / insect identification by deep learning exist, they do not often consider the broad generalization capability, which is critically important when these methods are rolled out for real public surveillance. This study successfully mitigate the performance drop caused by the difference of capturing devices and showed some conditions where generalization is extremely difficult. I think these results help researchers design effective strategies for machine learning-based vector surveillance.

The experiments are carefully designed to show the advantages and limitations of their method. The model architecture is standard and I do not find critical flaws in methodological aspects. The presentation of the manuscript is good. All parts are concisely presented and results are sufficiently discussed. Therefore, I think this manuscript in good quality, and I only have minor points for revision.

My only concern is the scope of the manuscript. I feel it is slightly more application-oriented than standard PLOS Computational Biology articles.

We thank the reviewer for their encouraging and insightful comments.

Minor points:
Line 117: Please report information of the capturing devices, for example, proportions of different devices.

We added information on the capturing devices in line 144-147

Line 338: Adding a UMAP visualization of trials without applying the pipeline may help show the effectiveness of the current methods. I guess that the clusters will separated based both on species and devices if the pipeline is not applied.

While such visualizations can be insightful, we chose not to include them due to the inherent variability of UMAP projections. As they rely on stochastic initialization and local optimization the resulting cluster structure can vary between runs, making it difficult to draw robust conclusions. To add our concern regarding the UMAP interpretation we modified the manuscript in line 419-420.

Line 416 image "angle"?

We are not quite sure what this comment refers to.

Line 423: Application of out-of-distribution detection methods may be a future direction.
Yang et al. 2024 <https://link.springer.com/article/10.1007/s11263-024-02117-4>
It may improve model performance since a large portion of errors are due to errors over the "other" category.

We agree that out-of-distribution detection represents a promising avenue for enhancing the robustness of our classification system. In fact, we have already conducted some experiments in this area and may want to explore these methods more systematically in future work. We have cited the recommended reference and noted this as a future direction in the manuscript in line 458.

Figure 5: "Level D" is missing in the caption of this figure (line 303). Also, captions are a bit confusing. Does "level D" include data augmentation or does it apply only image processing without augmentation?

Thank you for spotting this oversight. We have revised the caption of Figure 5 to clearly define “Level D” and to specify whether data augmentation was included. We have updated the image caption to provide clarity in line 339-350.
