# Supplementary material for: Potentials and limitations in the application of Convolutional Neural Networks for mosquito species identification using wing images
Source: PLoS Comput Biol. 2025 Sep 5;21(9):e1013435. doi: 10.1371/journal.pcbi.1013435 (PMC12412992; doi:10.1371/journal.pcbi.1013435)
Supplement: Attachment — Submitted filename: Respone to Reviewers_2107.docx [file pcbi.1013435.s003.docx]

Dear Zhaolei Zhang, dear team of PLOS Computational Biology,

Thanks for the invitation to respond to the reviewer comments. Below you find all comments in grey and our author response in **black**. In the revised manuscript all new changes are indicated in yellow.

With kind regards

Kristopher Nolte

**Reviewer #1**: I thank the authors for their point-by-point response to my previous comments. However, I’m afraid the revisions do not fully address the issues raised. The first two points below are particularly important, given the authors’ stated aim in the introduction to advance their previous work toward “practical, real-world deployment”:

1. Code Usability:
I appreciate the effort to update the instructions on the GitHub repository. However, it remains unclear how to run the code on a novel test image. For instance, if I capture an image of a mosquito wing using my phone, how do I test the classifier on this image? The README lists several Jupyter notebooks, but these lack detailed documentation. The test_classifier.ipynb notebook appears most relevant, yet it seems to require .npy files that have already been preprocessed. Clear, step-by-step instructions for running the model on raw images would be essential for real-world usability.

We split the work into two repositories but as we did not explain this in the manuscript this might be confusing. One was showcasing the installable docker application, where a user can upload a single or multiple raw images (<https://github.com/KNolte19/MosquitoWingClassifier>) for testing. The easiest way to test the application however is to go to <https://balrog.bnitm.de> log in with the credentials (login: balrog, password: PossiblyDoSurprisedWall, we will remove the password protection after publication) here you can upload images and get a prediction without installing the system yourself.

The other repository (<https://github.com/KNolte19/MosquitoWingClassifier_publication>) contains information how to replicate the training and analysis of the model. To further expand the documentation, we added a header with a brief description of the script and its input and outputs to every script.

We see that we did not really highlight the points mentioned here in our manuscript and acknowledge that this can be confusing, for this reason we added the following section to the manuscript.

Line 117: The final application is published in a GitHub repository ([GitHub Repository](https://github.com/KNolte19/MosquitoWingClassifier)) and is hosted as web-service on <https://balrog.bnitm.de>. All code to reproduce the results and figures in this manuscript can be found in second repository ([GitHub Repository](https://github.com/KNolte19/MosquitoWingClassifier_publication)).

Furthermore, to allow for the simple testing of raw images in code we added a script *utils/run_classifier_DEMO.ipynb* to the MosquitoWingClassifier_publication repository which let the user simply test new images using a notebook.

2. Feasibility Study and Benchmarking:
Section 4.4 remains limited and lacks accompanying figures. For example, including confusion matrices—one for a “known” imaging device and one for a “novel” device—would greatly enhance clarity. Additionally, there is no comparison with previously published models. Since the authors claim that "Traditional ML pipelines, while effective in many scenarios, frequently fall short in real-world deployment", it would be valuable to see how their model performs relative to the current state-of-the-art.

We agree that an additional figure would help to improve the clarity of the results. We modified this section and added confusion matrix to visualise the results. We also added the following sentence to highlight the low variance in performance we observed.

*Line 409: For the known device, accuracy ranged from 94.1% (worst) to 97.7% (best) across participant-model combinations. For the novel device, it ranged from 94.3% to 96.6%.*

Figure 8: Confusion matrix presenting the results of the feasibility study. On the left performance from 4 different participants and 5 models were averaged. Images were taken on a device for which the models were trained for. On the right performance for 5 models were averaged. Images were taken on an imaging device which was not represented in the training set.

The statement regarding “Traditional ML pipelines” was based on the publication (https://www.jmlr.org/papers/volume23/20-1335/20-1335.pdf) which investigated the issue of underspecification and demonstrated convincingly that Deep Learning models are often specified for their training domain and fail to produce reliable results in the real world due to not being generalizable to other changing conditions (e.g. different imaging device). As we had observed the same behaviour for our models in a previous publication (<https://parasitesandvectors.biomedcentral.com/articles/10.1186/s13071-024-06459-3>), we wanted to address this issue. Additionally research on tick species identification (<https://doi.org/10.1038/s41598-025-10265-x>) and general insect identification (<https://doi.org/10.1111/syen.12583>) reported similar issues.

We added references in line 81, 91, 440 to further strengthen our argument.

To test whether our methods were successful we conducted the robustness experiments shown in Fig. 5. While we agree that comparison with an external model would ideally strengthen the argument, the field of insect wing classification using CNNs remains narrow. The only other comparable work known to us besides our own (<https://doi.org/10.1038/s41598-024-53631-x>) is from Cannet et al. (https://www.nature.com/articles/s41598-025-08667-y) which focus on Wing Interference Patterns, using different features that are not directly transferable to our pipeline. We outperform our own models and their models on mosquito wings, yet the comparison of performance is difficult as it is extremely depended on the used species, and a benchmarking dataset is not available. Additionally, both previous studies adhered to a strictly standardized image collection process, which we sought to avoid.

With the aim of demonstrating the generalization capability of our approach, we conducted a structured evaluation using our own data and a set of robustness experiments. These experiments systematically compared models trained under varying levels of preprocessing — with the earlier stages representing a more “traditional” CNN pipeline, and later stages incorporating more preprocessing techniques. We found that models trained with minimal preprocessing were more vulnerable to real-world deployment challenges (Fig. 5): they struggled to generalize across imaging devices and were more susceptible to learning spurious correlations introduced by dataset composition. In contrast, models trained after applying the full preprocessing pipeline showed greatly improved robustness across both experimental setups.

While not a direct benchmark against external models, we believe these results provide meaningful evidence that the preprocessing plays a critical role in improving real-world reliability, offering context for the claim about the limitations of traditional pipelines in deployment scenarios.

3. Table Listing Data Used:
It is unclear where the supplementary information referenced by the authors can be found. I previously asked to see a breakdown of how exactly the data was used for training, testing, validation, etc. in the form of a table. The authors have advised that I should refer to the Git repository, but I'm not sure what exactly I should be referring to. I have also been referred to Supplementary Table S4, but I could not locate any supplemental information. What I would like to see is something similar to Tables 1 and 2 here, for example: <https://www.life-science-alliance.org/content/7/1/e202302351/tab-figures-data>.

We improved the description on where to find the metadata in the repository and added the following sentence to the manuscript:

*Line 164: “Comprehensive details about the images, including their labels, capture devices, and sources, are provided in the publications' GitHub repository (GitHub Repository) with a complete table describing all data (GitHub Repository/database_reference.xlsx).*

Furthermore, we provided an additional table as the reviewer suggested as table 1 in section 4.1, line 330.

Table 1: Composition of training and testing data in addition to performance results of each model.

| Testing Fold | N images used for Testing | N images used for Training | % images used for Training | Accuracy | Balanced Accuracy | Macro F1 Score |
| --- | --- | --- | --- | --- | --- | --- |
| 1 | 2469 | 12419 | 83.4 | 97.9 | 97.9 | 96.7 |
| 2 | 2486 | 12402 | 83.3 | 98.7 | 98.6 | 98.2 |
| 3 | 2439 | 12449 | 83.6 | 98.3 | 98.4 | 98.1 |
| 4 | 2492 | 12396 | 83.3 | 98.1 | 97.9 | 97.5 |
| 5 | 2437 | 12451 | 83.6 | 98.4 | 98.1 | 97.6 |

The supporting information can be found in the preceding submission at page 45, and in this submission on page 47.

We upgraded the documentation of both repositories to improve the clarity where relevant data sources and scripts can be found. We agree with the reviewer that this aspect of the work needed improvement, and we are thankful for time and effort the reviewer put in to improve the usability of our application as this is our stated goal.

**Reviewer #2:** This is a revised version of the manuscript I reviewed. It reports a preprocessing pipeline for mosquito identification based on wing images.

The authors responded most of the points I raised in the previous review round. Also, they made substantial revisions on the texts and added a new analysis. In the last review, I found the manuscript was already in good quality. The current version is better in quality with added explanations of backgrounds and a new feasibility study, which shows the generalization capability of the model.

I only have very minor points to mention over the current version.

Minor point:
Line 450 (Line 416 in the previous version): "image ANGLE" is spelled as "image ANGEL".

Thank you for spotting this oversight we fixed it in line 475.

*Line 475 “Furthermore, the depiction of whole mosquitoes can vary significantly due to factors such as age, physiological status (e.g. blood-fed or gravid), storage conditions, and image angle introducing additional variability that complicates the classification process (12,41)”*

GitHub repository:
Just like the reviewer 1, I encountered several errors before the codes properly ran. I think better documentations can lead to wider acceptance of this method by general users.

As mentioned already to first reviewer we are grateful for the effort put into checking our code to make it as useable as possible. We upgraded the documentation of both repositories to make clear where relevant data sources and scripts can be found. If any further errors occur or some feedback for the documentation comes up, please also feel free to contact us directly so we can fix the issue at hand. Thank you.
